# Orbital Eccrine Hidrocystoma

**DOI:** 10.4274/tjo.98853

**Published:** 2016-10-17

**Authors:** Deniz Marangoz, Işın Doğan Ekici, Ferda Çiftçi

**Affiliations:** 1 Yeditepe University Faculty of Medicine, Department of Ophthalmology, İstanbul, Turkey; 2 Yeditepe University Faculty of Medicine, Department of Pathology, İstanbul, Turkey

**Keywords:** Eccrine gland, hidrocystoma, orbital cystic mass

## Abstract

A 29-year-old female patient presented with a painless mass on her upper eyelid medially. She noticed the mass 4 years earlier and it had increased in size over time. She had no diplopia, eyelid swelling, skin lesion overlying the mass, or visual disturbances. On ocular examination, eye movements and funduscopy were normal. The mass was movable and painless with palpation. Magnetic resonance imaging with contrast showed a 12x8x7 mm well-circumscribed cystic lesion with no contrast dye appearance. Surgical removal was performed delicately and no capsular rupture occured. Pathological examination revealed an eccrine hidrocystoma. Our aim is to underline that eccrine hidrocystoma should be included in differential diagnosis of orbital masses.

## INTRODUCTION

Hidrocystoma is a rare benign cutaneous cystic lesion originating from sweat glands (eccrine or apocrine). Hidrocystoma occurs predominantly in the head and neck region. Solitary lesions occur with equal frequency in both genders, while multiple lesions are more common in women.

Eccrine hidrocystomas may manifest as single or multiple small (1-6 mm in diameter), thin-walled cysts.^1^ Lesions in the head/neck region are most often located in the periorbital/malar area.^2^ Apocrine hidrocystomas are usually solitary and 3-15 mm in size.^1^ In this case report we aimed to highlight that orbital eccrine hidrocystoma, though rare, should be included in the differential diagnosis of orbital lesions.

## CASE REPORT

**Figure 1:**
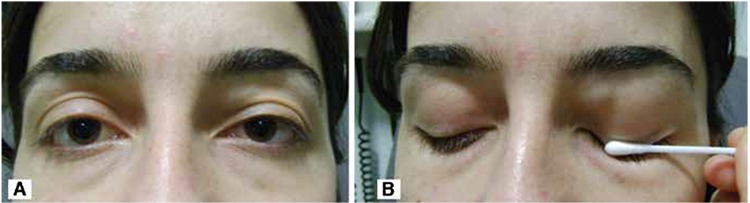
A and B. External photograph showing mass in the medial left upper eyelid

A 29-year-old female patient presented to our clinic with complaints of a mass located medially on her left upper eyelid which had increased in size over the previous 4 years (Figure 1). The mass was movable and painless on palpation. The patient had no diplopia, and there was no erythema or warmth in the skin overlying the mass. Visual acuity was 20/20 in both eyes on Snellen chart, and intraocular pressure as measured by noncontact tonometry was within normal limits. The patient’s eye movements were unrestricted. Direct and indirect light reflexes were normal. No pathology was detected on the skin over the mass on slit-lamp examination. Anterior segment structures and fundus examination were normal in both eyes.

**Figure 2:**
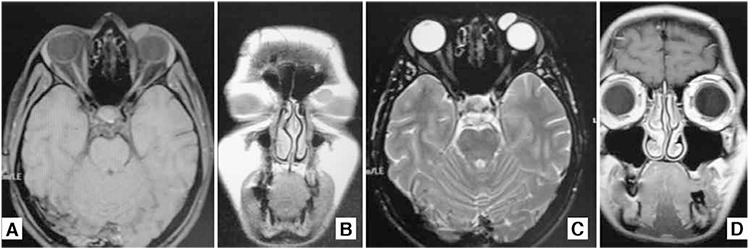
Magnetic resonance imaging of the mass: A Mild hyperintensity on T1-weighted horizontal section; B. Marked hyperintensity on T2-weighted horizontal section; C and D. In coronal section the mass is visible superomedially adjacent to the left globe

The patient first noticed the mass 4 years earlier and since then its growth was documented by annual follow-up with orbital magnetic resonance imaging (MRI) with contrast (Figure 2 shows orbital MRI of the cystic mass from January 2014). It was documented as a dense cystic mass lesion with smooth borders, approximately 12x8x7 mm in size, located anteromedially to the left eye. It appeared mildly hyperintense on T1-weighted images and markedly hyperintense on T2-weighted images, and showed no enhancement pattern.

**Figure 3:**
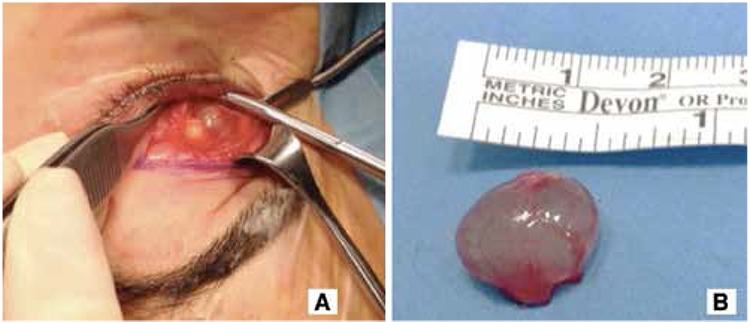
Surgical removal of the mass. A. The cystic mass, located behind the septum, was exposed by cutaneous incision in the medial upper eyelid and blunt dissection; B. The cystic mass excised as a whole with capsule intact

**Figure 4:**
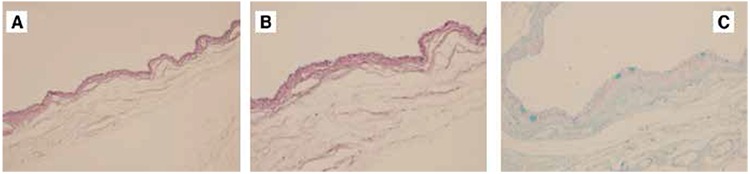
Histopathological examination of the mass. A. Single-layer cuboidal epithelium, hematoxylin&eosin (H&E) x100; B. H&E x200; C. Positive cytoplasmic staining of the sporadic goblet cells in the lining epithelium

To surgically excise the mass, a cutaneous incision was made medially in the fold of the left upper eyelid and the mass was exposed by blunt dissection through the orbicular muscle and septum. Excision was continued by careful blunt dissection in order to avoid capsule rupture and the cystic mass was removed from the surrounding tissues. Following the total excision of the mass (Figure 3), it was sent for pathologic analysis. Macroscopically the mass was 15x15x6 mm in size and whitish-pink in color with translucent margins; microscopically the mass was determined to be a benign cystic formation lined with a single layer of columnar epithelium. Immunohistochemically, the mass did not exhibit ki-67 staining, while histochemical staining with Alcian blue revealed the presence of sporadic goblet cells in the mass epithelium (Figure 4). The results of the pathologic analysis reported that the mass was consistent with eccrine hidrocystoma.

## DISCUSSION

Hidrocystomas in the periorbital region are often located in the eyelid and inner canthus.^3^

Apocrine sweat glands are limited to specific regions like the axilla, nipple, external ear, external genitalia and eyelids, while eccrine sweat glands are widely distributed across the body.^4^

Eccrine hidrocystoma was first described by Robinson^5^ and was classified according to lesion number as Robinson (multiple) or Smith (solitary) type. Eccrine hidrocystomas generally expand in summer and spontaneously regress in cooler weather.^1^

Hidrocystomas do not recur if total excision is achieved. Because eccrine hidrocystomas are usually subcutaneous, they are obvious and can be diagnosed even at very small sizes (1-6 mm).^1^ Our patient had a mass with a maximum diameter of 15 mm; it was likely able to reach this size because it developed over 4 years within the orbit.

Other cystic lesions included in the differential diagnosis of eccrine hidrocystomas are follicular cysts, epidermal inclusion cysts, hemangioma, lymphangioma, apocrine hidrocystoma and eccrine acrospiroma.

Cases of ‘orbital apocrine hidrocystoma’ have been reported in the literature by Valenzuela and Heathcote^6^, Ssi-Yan-Kai and Pearson^7^, Vignes et al.^8^ and Mehta et al.^9^

Valenzuela and Heathcote^6^ described a 47-year-old male patient with a 3-month history of a painless mass in the superomedial aspect of his left upper eyelid which was impinging on his peripheral visual field. Orbital computed tomography (CT) imaging revealed a 13x8 mm extraconal cystic mass in close proximity to the globe. Mass excision by anterior orbitotomy was planned, but due to the attachments of the mass to the superomedial orbit, the brown contents of the cyst were first removed, then the excised cyst wall was sent for histopathologic examination. The inner walls of the cyst were lined with columnar epithelium and the cells contained PAS (Periodic Acid Schiff)-positive material in the apical cytoplasm. The mass was determined to be consistent with orbital apocrine hidrocystoma.

In a case reported by Ssi-Yan-Kai and Pearson^7^ a 46-year-old woman had soft mass growing on the medial aspect of her right lower eyelid for several months. The mass had displaced the globe superolaterally, but the patient’s eye movements appeared normal. Orbital CT revealed an 18 mm mass. However, the cyst ruptured during surgical excision and was determined insufficient for histopathological examination. Two years later, orbital MRI performed due to recurrence revealed another 17.5 mm cystic mass, also located in the inferomedial aspect of the orbit, which was surgically excised with cyst capsule intact. On histopathological examination, the mass was reported as ‘benign apocrine hidrocystoma’.

Vignes et al.^8^ reported the case of a 33-year-old male with an 18-month history of progressive swelling of his right eyelid. There was no pathology apparent in the eyelid other than edema. Visual acuity and eye movements were normal. An intraorbital extraconal cystic lesion was detected on orbital MRI. The mass was surgically excised and diagnosed as ‘apocrine hidrocystoma’ based on histopathological examination.

Mehta et al.^9^ described a 65-year-old woman who presented complaining of ptosis starting 10 days earlier and a mass in her left upper eyelid. The patient had undergone eyelid repair 7 years earlier due to traumatic ptosis, but no ptosis or mass had been reported in an ocular examination 2 months earlier. Eye movements were restricted on upward gaze and levator function was 14 mm on the right and 5 mm on the left. A soft mass 1.5 mm in diameter could be palpated at the medial aspect of the left supraorbital margin. Orbital CT and ultrasonography were performed and the cystic mass was completely excised after dissecting its adhesions to the levator aponeurosis. Histopathologic examination was consistent ‘apocrine hidrocystoma (sudoriferous cyst)’. The authors attributed cyst development in this patient to one of two mechanisms: either superficial sweat gland cells were implanted in deeper tissues at the time of the injury, or some epithelial cells were implanted into deeper tissues during the eyelid surgery following the trauma, where they gradually proliferated to form a cyst.

To our knowledge, there are no reports of orbital eccrine hidrocystoma in the literature other than the case of a 14-year-old patient with giant eccrine hidrocystoma reported by Eslami et al.^10^ That case involved a 3-month history of a painless mass in the right upper eyelid. The patient presented with diplopia, proptosis and ptosis. Orbital CT revealed a 1.5x1 cm oval mass in the superomedial aspect displacing the right globe inferolaterally. Total excision was performed by cutaneous incision. The mass was a giant eccrine hidrocystoma, 1x1x2.5 cm in size, encapsulated in fibrous connective tissue.

Our aim in this study was to emphasize that eccrine hidrocystomas, although rare, should be considered during differential diagnosis of orbital masses.

### Ethics

Informed Consent: It was taken.

Peer-review: Externally peer-reviewed.
